# (Poly)phenols and brain health – beyond their antioxidant capacity

**DOI:** 10.1002/1873-3468.14988

**Published:** 2024-07-23

**Authors:** Thomas Hunt, Matthew G. Pontifex, David Vauzour

**Affiliations:** ^1^ Norwich Medical School University of East Anglia Norwich UK

**Keywords:** Alzheimer's disease, cognition, flavonoids, gut‐brain axis, microbiota

## Abstract

(Poly)phenols are a group of naturally occurring phytochemicals present in high amounts in plant food and beverages with various structures and activities. The impact of (poly)phenols on brain function has gained significant attention due to the growing interest in the potential benefits of these dietary bioactive molecules for cognitive health and neuroprotection. This review will therefore summarise the current knowledge related to the impact of (poly)phenols on brain health presenting evidence from both epidemiological and clinical studies. Cellular and molecular mechanisms in relation to the observed effects will also be described, including their impact on the gut microbiota through the modulation of the gut‐brain axis. Although (poly)phenols have the potential to modulate the gut‐brain axis regulation and influence cognitive function and decline through their interactions with gut microbiota, anti‐inflammatory and antioxidant properties, further research, including randomised controlled trials and mechanistic studies, is needed to better understand the underlying mechanisms and establish causal relationships between (poly)phenol intake and brain health.

## Graphical abstract

Dietary (poly)phenols are of great interest as they have been demonstrated to reduce cognitive decline. Past research has focused on their role as antioxidant molecules; however, it has now been established that relatively low concentrations reach the cerebral tissue. This Review investigates the literature surrounding alternative mechanisms of actions of (poly)phenols from a variety of different dietary sources.

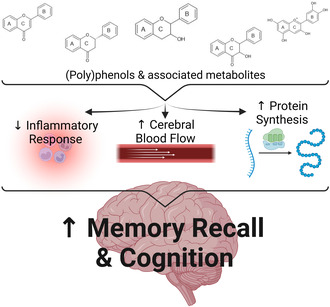

## Abbreviations

3C, Three Cities

AD, Alzheimer's disease

ADAS‐Cog, The Alzheimer's Disease Assessment Scale–Cognitive Subscale

ADME, absorption, distribution, metabolism, and excretion

ANS, autonomic nervous system

AOE, antioxidant enzyme

ARE, antioxidant response element

BDNF, brain‐derived neurotrophic factor

CBF, cerebral blood flow

CDR, clinical dementia rating

CNS, central nervous system

COX2, cyclooxygenase‐2

CREB, cAMP response element‐binding protein

DOPAC, 3,4‐dihydroxyphenylacetic acid

ENS, enteric nervous system

ERK, extracellular signal‐regulated kinase

FFQ, food frequency questionnaire

GBA, gut‐brain axis

HPA, the hypothalamic–pituitary–adrenal axis

IFN, interferon

IL, interleukin

iNOS, inducible nitric oxide synthase

JNK, c‐Jun N‐terminal kinases

LMW, low molecular weight

LPS, lipopolysaccharide

MAPK, mitogen‐activated protein kinase

MCI, mild cognitive impairment

MMSE, mini mental state examination

NF‐kB, nuclear factor kappa‐light‐chain‐enhancer of activated B cells

PCA, protocatechuic acid

PDE, phosphodiesterase

PKC, protein kinase C

RR, relative risk

SCFA, short‐chain fatty acid

SIRT1, silent mating type information regulation 2 homologue 1

TLR, toll‐like receptor

TMA, trimethylamine

TNF, tumour necrosis factor

**Fig. 1:**
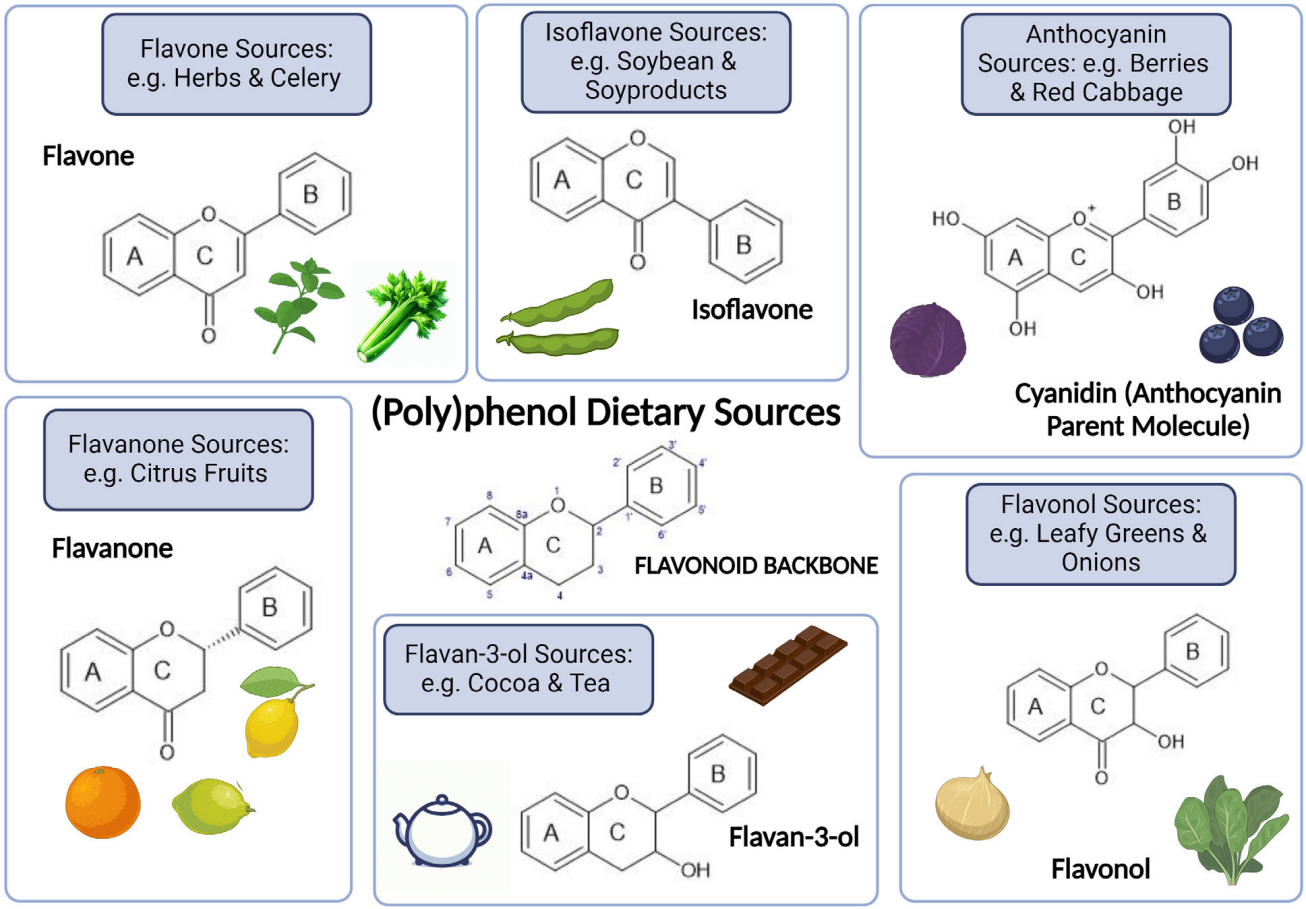
A list of the six subclasses of flavonoids and some of their example dietary sources.

Dementia is expected to double every 20 years, reaching 152 million by 2050 [1, 2]. Despite intensive research and drug development activities, there remains only limited drug therapies available to prevent, delay and ameliorate the progression of neurodegenerative disorders. As a result, there is a wide interest in identifying lifestyle strategies, such as nutritional interventions, to reduce disease risk and mitigate dementia prevalence [3, 4]. Numerous human observational studies highlight the benefits of adhering to a diet rich in plant‐based foods, with dietary (poly)phenol intake demonstrated to be particularly influential upon cognitive function [5, 6, 7, 8].

(Poly)phenols are a large and heterogeneous group of secondary metabolites found in various fruits and vegetables along with beverages such as tea, coffee and red wine. Epidemiological studies have estimated that the total (poly)phenol intake in Europe ranges from 584 to 1786 mg·day^−1^ *per capita* [9]. Structurally, these natural compounds are classified into flavonoids and non‐flavonoids [10]. Flavonoids are characterised by a C6‐C3‐C6 structure and can be further subdivided based on their chemical complexity into six distinct subgroups: flavan‐3‐ols, flavonols, isoflavones, anthocyanins, flavones and flavanones (Fig. 1). Non‐flavonoids comprise phenolic acids, lignans, coumarins and stilbenes [11]. These compounds are produced by plant metabolism and do not usually exist in free‐from, instead the majority are bound to other molecules such as glycosides or esters of glucose, tartaric acid and quinic acid [11]. Over the past decades, there have been significant advances in the field of (poly)phenols and brain health, encompassing their potential application in neurodegenerative disorders. Amongst the most established mechanistic actions, (poly)phenols have been reported to reduce neuroinflammation, improve synaptic plasticity, increase cerebral blood flow, counteract mitochondrial dysfunction, and mitigate proteinopathies/excitotoxicity [12]. However, such processes vary according to the (poly)phenol source, dosage and bioavailability [12]. Indeed, only a small fraction of ingested (poly)phenols is absorbed in their original form due to their chemical structure, solubility, and the presence of other dietary components. Following limited absorption in the small intestine, (poly)phenols will primarily be modified in the liver into Phase I (oxidation, reduction, hydrolysis) and Phase II (conjugation with glucuronic acid, sulfate, or methyl groups) metabolites. These hepatic metabolites may enter the systemic circulation and reach the brain as they are more bioavailable than their parent compounds. (Poly)phenols that are not absorbed in the small intestine will reach the colon, where they will be catabolised by the gut microbiota into smaller phenolic acids and other metabolites, which will subsequently enter the bloodstream and eventually cross the blood–brain barrier and enter the brain [10, 11, 13]. Given these developments and our growing understanding of (poly)phenols in the context of brain health, there is now considerable interest in improving the translational potential of these molecules (e.g., establishing combinations/dosage and tailoring to disease). This review will, therefore, summarise the current knowledge related to the impact of (poly)phenols on brain health, presenting evidence from both epidemiological and clinical studies. Cellular and molecular mechanisms in relation to the observed effects will also be described, including their impact on the gut microbiota.

## (Poly)phenol intake, cognitive decline and dementia: epidemiological evidence

Epidemiological studies investigating the impact of (poly)phenols on brain function have gained significant attention due to the growing interest in the potential benefits of these dietary bioactive molecules for cognitive health and neuroprotection. These studies have utilised various methodologies, including dietary assessments, cognitive tests and longitudinal follow‐ups. Cognitive benefits have been in large part attributed to specific (poly)phenols [14] and (poly)phenol‐rich foods and beverages [8, 15]. In particular, positive associations between increased consumption of total flavonoids and episodic memory were reported in middle‐aged adults, and higher intakes of berries were associated with a reduced rate of cognitive decline in adults aged 70 and over [16]. Furthermore, in a 4–5 years follow‐up, daily consumption of green tea, but not coffee or black tea, was associated with a reduced risk of cognitive decline as assessed by the Mini‐Mental State Examination (MMSE) and Clinical Dementia Rating (CDR) [17]. Similarly, in a cross‐sectional study, consumption of flavan‐3‐ol‐rich chocolate, wine, or tea significantly improved cognitive performance in a dose‐dependent manner in older men and women [18]. Furthermore, in a prospective study of 1329 participants of the Three Cities (3C) Bordeaux Cohort with a 12‐year follow‐up, participants in the higher quintile of (poly)phenol intake had a 50% lower risk of dementia (95% CI 20%; 68%, p‐trend < 0.01) compared to low consumers [7]. Results from the Framingham Heart Study Offspring Cohort also reported that higher long‐term dietary intakes of flavonols, anthocyanin and flavonoid polymers were associated with lower risks of Alzheimer's disease (AD)‐related dementia and AD in US adults [19]. Finally, a recent systematic review and meta‐analysis of observational studies has reported an inverse association with cognitive impairment and reduced association with the incidence of dementia or related disorders for total flavonoids [relative risk (RR) = 0.83, 95% CI: 0.76, 0.89]. In particular, anthocyanins (RR = 0.73, 95% CI:0.60, 0.89), flavones (RR = 0.77, 95% CI: 0.63, 0.94), flavan‐3‐ols (RR = 0.86, 95% CI: 0.82, 0.91) and flavonols (RR = 0.88, 95% CI: 0.80, 0.96) appeared to be the most beneficial [20]. Although these prospective cohorts provide an initial indication of cognitive benefits associated with a (poly)phenol‐enriched diet, their use of dementia screening tests such as the MMSE [21, 22, 23] and/or the ADAS‐Cog or the CDR [24] to assess global cognitive abilities rather than full conventional battery tests, means that they do not provide insights into effects on specific memory types. Furthermore, while epidemiological studies provide valuable insights into the potential relationship between (poly)phenol intake and brain function, it is essential to consider the limitations of such studies, for example, dietary assessment methods used, confounding factors, and the possibility of reverse causation.

Indeed, (poly)phenol intakes are usually self‐reported and strongly influenced by the number of food items included in food frequency questionnaires (FFQ). Moreover, using different reference databases, many of which are very limited in estimating the (poly)phenol content of foods, may affect the estimation of (poly)phenol content in foods [25]. Additionally, the diverse nature of (poly)phenols and their varying bioavailability and metabolism make it challenging to establish causal relationships based solely on observational studies [10]. Despite these challenges, epidemiological evidence suggests that higher intake of (poly)phenol‐rich foods may be associated with better cognitive function and a reduced risk of neurodegenerative diseases.

## Impact of (poly)phenol interventions on human cognition: clinical evidence

Human intervention studies investigating the impact of (poly)phenols on cognition have gained significant attention in recent years. These studies involve administering (poly)phenol‐rich foods, extracts, or supplements to human participants and assessing their cognitive function through various measures. Several of these studies have yielded promising results, suggesting that (poly)phenols may have beneficial effects on cognition. For example, a recent meta‐analysis of randomised controlled trials investigating flavonoid effects on human cognition and comprising 5519 participants, has reported that (poly)phenol‐rich food and in particular cocoa (*g* = 0.224, *P* = 0.036), ginkgo (*g* = 0.187, *P* ≤ 0.001) and berries (*g* = 0.149, *P* = 0.009) have significant positive effects on long‐term memory, processing speed and mood in middle‐aged and older adults [26]. Amongst the flavonoids, flavan‐3‐ol interventions at doses above 520 mg·day^−1^ and over a period of more than 8 weeks have shown improvements in cognitive tasks. However, only a few cognitive domains were targeted in such trials [21, 27, 28, 29]. On the other hand, doses lower than 500 mg·day^−1^ have been less successful at achieving neurocognitive benefits [30, 31, 32, 33, 34], although a study found an improvement in working memory and executive function in young adults (22–24 years) at intakes of 500 mg·day^−1^ [35]. This result may be attributable to the longer treatment period of 2 months [35], with other studies, intervening either acutely or for periods ranging from 5 to 30 days [30, 31, 32, 33, 34]. It is noteworthy that some studies described improvements in cerebral blood flow (CBF) parameters [32] or activation of brain regions when performing a cognitive task (but no behavioural effects) using either a steady state probe topography [31] or functional magnetic resonance imaging [28, 30, 36]. Maintaining CBF is essential to allow the supply of nutrients and oxygen to cells and to remove toxic waste products produced during cell metabolism. CBF has been shown to gradually decline as an individual ages and is linked to neurocognitive decline and a marked decline in dementia patients [37]. Varying (poly)phenol sources (cocoa, cranberry and epigallocatechin gallate) have been reported to increase CBF [32, 38] leading to improved cognitive outcomes. For example, acute flavan‐3‐ol‐rich cocoa intake (8.3 g per individual dose) was reported to improve cognitive function and efficiency in blood oxygenation in frontal cortical areas of young healthy subjects during hypercapnia [39]. In addition, flavan‐3‐ol rich chocolate (635 mg) was reported to enhance the efficient use of cognitive resources and to reduce the cost of brain activity during continuous and effortful tasks [40]. Similarly, chronic consumption of flavan‐3‐ol‐rich cranberries (*Vaccinium macrocarpon*) for 12 weeks improved episodic memory and regional brain perfusion in healthy older adults (50–80 years) [38]. Interestingly, a recent study reported that older adults with lower diet quality and lower habitual flavan‐3‐ol consumption showed lower hippocampal‐dependent memory, suggesting that low flavan‐3‐ol consumption may also act as a driver of cognitive ageing [41].

Using a higher dose (approximately 900 mg·day^−1^) for a shorter period of treatment (6 weeks) did not result in improvements in either episodic and recognition memory or executive function [42]. These observations suggest that a treatment duration of at least 2 months and a dose greater than 520 mg·day^−1^ is necessary to exhibit beneficial cognitive impacts in human trials [21, 27, 28, 35]. However, caution should be taken regarding extrapolation and interpretation of the data as the trials published to date focus mainly on object recognition memory, semantic memory and executive function. Other domains, such as short‐term working memory and episodic memory, known to be hippocampal‐dependent and affected by age and age‐related neurodegenerative diseases, were rarely investigated.

Relative to flavan‐3‐ols, lower doses of anthocyanins have been shown to improve neurocognitive deficits. For instance, 226 mg·day^−1^ of anthocyanins provided as Concord grape juice [36, 43] over 12–16 weeks improved episodic memory in elderly healthy and mild cognitive impaired patients, although other studies did not observe an improvement following a higher acute intake (700 mg·day^−1^) of Concord grape juice in younger adults (18–50 years) [44]. Only implicit memory was assessed in these studies; therefore, the impact of intervention on other cognitive domains cannot be precluded. Similarly, an acute dose of blueberry in children aged 8–10 years failed to provide a beneficial effect on working memory and executive function [45]. However, healthy older individuals aged 65–80 years receiving 26 g of freeze‐dried wild blueberry powder (302 mg anthocyanins) showed enhanced immediate recall on the auditory verbal learning task along with better accuracy on a task‐switch task [46]. Furthermore, chronic consumption (6 months) of a (poly)phenol‐rich extract from grape and blueberry (600 mg·day^−1^, 235 mg flavonoids) improved visuospatial learning and episodic memory in a subgroup of healthy elderly subjects (60–70 years old) with advanced cognitive decline [47]. Additionally, anthocyanin‐rich *Aronia melanocarpa* extract (90 mg) for 24 weeks improved psychomotor speed in middle‐aged individuals at risk of cognitive decline [48] (Table 1).

**Table 1:** Overview of intervention studies on (poly)phenols in brain health and disease in humans.

Intervention	Dose and duration	Age	Health status	Results	References
Flavan‐3‐ols					
Flavan‐3‐ol rich cocoa drink	≈ 990 mg·day^−1^ 8 weeks	Elderly adults	Mild cognitive impairment	Improved verbal fluency	[21]
Flavan‐3‐ol rich cocoa drink	993 mg·day^−1^ 8 weeks	Elderly adults	Healthy	Improved cognitive function and verbal fluency	[27]
Flavan‐3‐ol rich cocoa drink	994 mg·day^−1^ Acute	Adults	Healthy	Acute improvements in mood and cognitive performance during sustained mental effort	[29]
Cocoa flavan‐3‐ol	150 mg·day^−1^ 5 days	Adults	Healthy	Increase the BOLD signal Intensity in response to an intense cognitive task	[30]
Cocoa flavan‐3‐ols	500 mg·day^−1^ 30 days	Adults (40–65 years old)	Healthy	No effect	[31]
Flavan‐3‐ol rich cocoa drink	500 mg·day^−1^ 30 days	Adults	Healthy	No effect	[33]
Flava‐3‐nol rich cocoa supplement	250 mg·day^−1^ 4 weeks	Adults	Healthy	Improved self‐reported mental fatigue	[34]
High flavan‐3‐ol diet	Not specified	Adults (50–69 years)	Healthy	Improved dentate gyrus function	[28]
Cacao rich dark chocolate	635 mg·day^−1^ Acute	Adults (30–49 years)	Healthy	Enhances the efficient use of cognitive resources by reducing the effort of brain activity	[40]
Cocoa flavan‐3‐ols	500 mg·day^−1^ 3 years	Older Adults	Healthy	Flavan‐3‐ol intervention improves the memory of those on the poorest diets of the cohort	[41]
Dark chocolate bar	37 g·day^−1^ 6 weeks	Adults (> 60 years)	Healthy	No effect	[42]
Epigallocatechin gallate	270 mg Acute	Adults	Healthy	Reduced CBF in the frontal cortex	[32]
Catechin‐rich oil palm leaf extract	0.5 g·day^−1^ 2 months	Young adult	Healthy	Improved short‐term memory	[35]
Cranberry supplementation	100 g equivalent of fresh berries per day 12 weeks	Adults (50–80 years)	Healthy	Improves episodic memory and regional brain perfusion	[38]
Flavonols and flavones					
Quercetin	1000 mg·day^−1^ 12 weeks	Adults (18–85 years)	Healthy	No effect	[53]
Ginkgo biloba extract	24% flavone glycosides, 6% terpene lactones and less than 5 ppm ginkgolic acids 6 weeks	Adults (> 60 years old)	Healthy	Improved SRT tasks involving delayed and WMS‐III FII subtest assessing delayed	[49]
Ginkgo biloba extract	240 mg·day^−1^ 6 weeks	Adults (45–56 years)	Healthy	Improved quality and quantity of recall	[50]
Gingko biloba extract	120 mg·day^−1^ 6 months	Adults	Alzheimer's disease patient currently prescribed cholinesterase inhibitors	Improvement in the mini mental state examination score in those taking the ginko extract	[51]
Ginkgo biloba extract	240 mg·day^−1^ 24 weeks	Adults	Mild to moderate dementia – Alzheimer's disease or vascular dementia	Neuropsychiatric inventory score improvement	[52]
Ginkgo biloba extract	240 mg·day^−1^ 22 weeks	Adults (> 50 years)	Alzheimer's disease patients with neuropsychiatric features	No significant effect	[54]
Ginkgo biloba extract	160 mg·day^−1^ 24 weeks	Adults (50–80 years)	Mild to moderate dementia	Improvement in the score of the clinical global impression score	[55]
Anthocyanins					
Wild blueberry juice	Daily consumption between 6 and 9 mL·kg^−1^ 12 weeks	Adults	Age‐related memory decline	Improved verbal paired associate learning test	[43]
Concord grape juice	Daily consumption between 6.3 and 7.8 mL·kg^−1^ 16 weeks	Adults (70–85 years)	Mild cognitive impairment	Reduced semantic interference on memory tasks	[36]
Flavonoid‐rich blueberry drink	143 mg anthocyanins Acute	Children (8–10 years)	Healthy	Improved the delayed recall of a previously learned list of words	[45]
Freeze‐dried blueberry	26 g freeze dried berries 12 weeks	Older adults	Healthy	Improved cognitive function	[46]
(Poly)phenols from blueberry and grape	258 mg·day^−1^ 6 months	Elderly adults (60–70 years)	Healthy	Improves age‐related episodic memory decline in individuals with the highest cognitive impairments	[47]
Grape juice	10 mL·kg^−1^ Acute	Adults (18–50 years)	Smokers	No effect	[44]
Anthocyanin‐rich *Aronia melanocarpa* extract	150 mg·day^−1^ 24 weeks	Adults (40–60 years)	Healthy	Improved psychomotor speed	[48]

Flavonols derived from Ginkgo biloba EGb761 extract, at doses between 120 and 240 mg·day^−1^, showed neurocognitive benefits in healthy adults [49, 50] or AD patients [51, 52]. However, it is noteworthy that the treatment period of 6–24 weeks was much longer than previously mentioned studies using between 172 and 250 mg·day^−1^ of cocoa flavan‐3‐ols [30, 31, 33, 34], highlighting the likely crucial role of the duration of intervention in human efficacy trials. In contrast, a flavonol dose of 500 or 1000 mg·day^−1^ over 12 weeks failed to observe a beneficial impact of quercetin in healthy middle‐aged patients [53]. The apparent lack of consistent benefits following (poly)phenol intervention might result from the participant's cognitive status at baseline, with the majority conducted in healthy individuals. Participants with mild cognitive impairment (MCI), mild to moderate AD or vascular dementia appear to show more pronounced cognitive benefits following (poly)phenol treatment for at least 12 weeks and up to 24 weeks [52, 54, 55]. Therefore, when conducting (poly)phenol interventions, careful consideration should be given to the participants' cognitive status and overall phenotype, along with their lifestyle habits and dietary intake. In particular, consideration should be given to the use of a high (poly)phenol intake as an exclusion criterion as these individuals are not representative of the general population, and a high baseline status is likely to result in a null response to intervention. Finally, the lack of consistent and accurate description of memory forms targeted and assessed, as well as the use of isolated rather than a panel of cognitive tasks, is likely to contribute to inaccurate interpretation of the results and to inter‐study inconsistencies. Some important memory domains which are impaired by ageing, as well as in pathological conditions such as MCI and AD, are under‐represented or not explored [56]. Conclusions using terms such as “neurocognitive benefits”, “improves memory function” or “rescues cognitive benefits” are often drawn based on only a few cognitive tests performed, while test batteries would provide a better overview of general cognitive function in response to intervention. Overall, human intervention studies provide compelling evidence for the potential cognitive benefits of (poly)phenols. However, it is important to note that further research is needed to understand the mechanisms underlying these effects and to determine the optimal doses and sources of (poly)phenols for better cognitive health (Table 1).

## Mechanisms of action

### (Poly)phenols as antioxidants or signalling molecules?

**Fig. 2:**
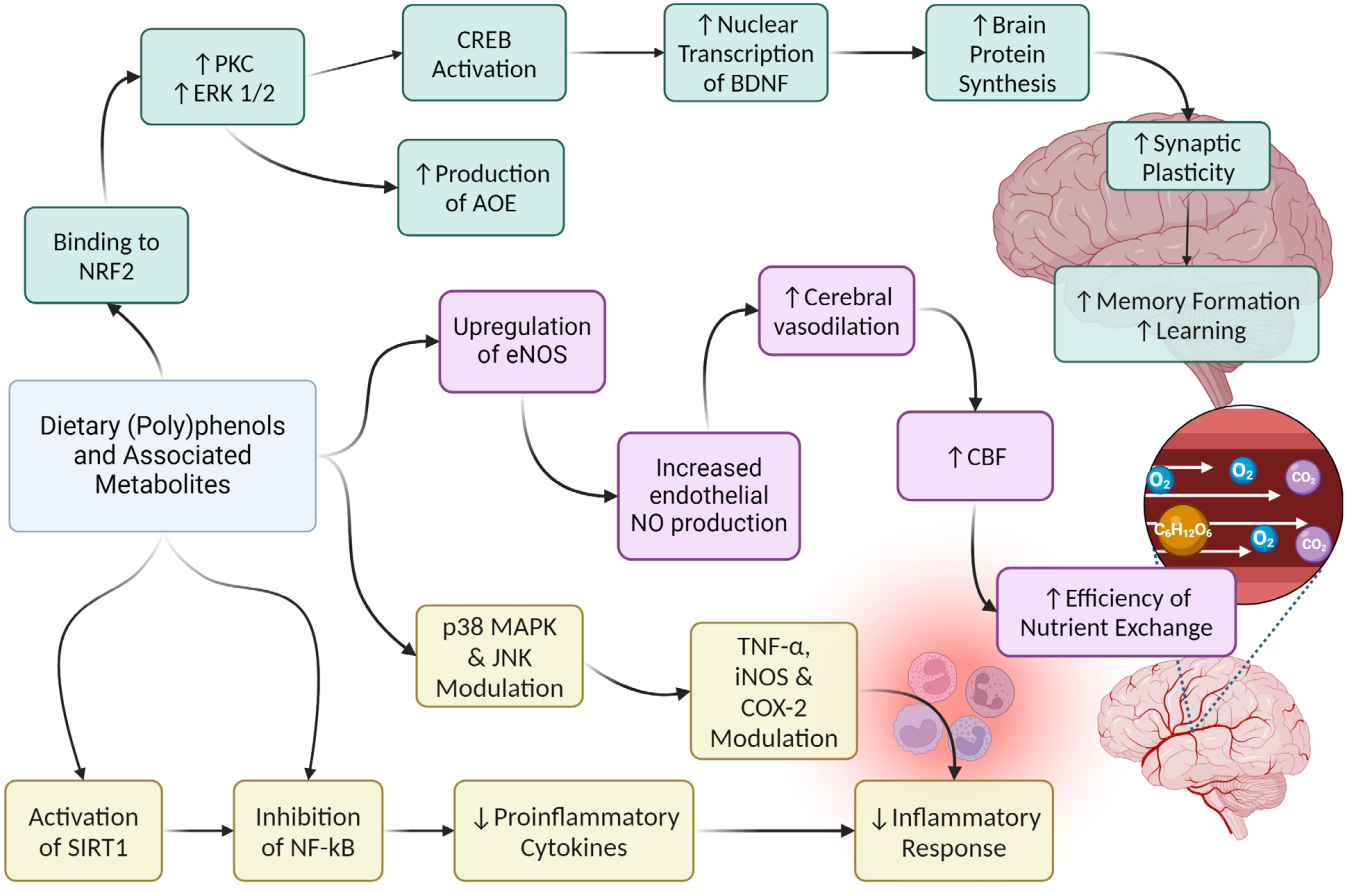
Mechanisms of dietary polyphenols and their metabolites on the brain and the immune response proposed for their observed effects on systemic health. The antioxidant response mechanism shows increased protein kinase C (PKC) and extracellular signal‐regulated kinase 1/2 (ERK 1/2) stimulation by binding to NRF2 and subsequent activation of antioxidant enzymes (AOE). Increased ERK 1/2 activation and CREB activation induce the nuclear transcription of the brain‐derived neurotrophic factor (BDNF), increasing the protein synthesis in the brain and the synaptic plasticity, improving learning and memory formation. Polyphenols also reduce the synthesis of pro‐inflammatory cytokines by inhibiting nuclear factor kB (NF‐kB) directly and indirectly by inhibiting protein sirtuin‐1 (SIRT1). Polyphenols also modulate p38 mitogen‐activated protein kinase (p38 MAPK) and Jun amino‐terminal kinases (JNK). These two kinases regulate the expression of tumour necrosis factor α (TNF‐α), inducible nitric oxide synthase (iNOS) and cyclooxygenase 2 (COX‐2).

(Poly)phenols have been posited as antioxidant agents due to their electron‐donor capacity from the phenolic groups [57, 58]. However, their plasma concentration is far lower than endogenous antioxidant molecules (e.g. glutathione, vitamin C and E, etc). Instead, it has been demonstrated that their protective activities could derive from an indirect modulation of cellular signalling pathways that mediate cell function in both physiological and pathological conditions [11, 13, 59]. For example, (poly)phenols and their metabolites can stimulate the antioxidant response element (ARE) that encodes cytoprotective proteins such as antioxidant enzymes (AOE). The activation of ARE is induced by the binding with Nrf2, which is promoted by (poly)phenols and their stimulation of the extracellular signal‐regulated kinase 1/2 (ERK 1/2) and protein kinase C (PKC) [60]. In addition, (poly)phenols have also been associated with the activation of ERK 1/2 and subsequent CREB activation, which induce the nuclear transcription of brain‐derived neurotrophic factor (BDNF) [61, 62, 63]. BDNF is an important factor that stimulates protein synthesis in the brain and synaptic plasticity, influencing thus learning and memory formation [64]. (Poly)phenols not only induce the expression of AOE and neurotrophic factors but also reduce the synthesis of pro‐inflammatory cytokines through the inhibition of the nuclear factor kB (NF‐kB) [65]. NF‐kB can also be inhibited via the indirect activation of the protein sirtuin‐1 (SIRT1) by (poly)phenols through phosphodiesterase (PDE) enzymes. Furthermore, (poly)phenols are able to modulate the p38 mitogen‐activated protein kinase (p38 MAPK) and Jun amino‐terminal kinases (JNK), two key protein kinases which regulate the expression of the tumour necrosis factor α (TNF‐α), inducible nitric oxide synthase (iNOS) and cyclooxygenase 2 (COX‐2) [66, 67, 68]. With these mechanisms, (poly)phenols block the production of reactive oxygen species, interleukin 1β (IL‐1β), nitric oxide (NO), thereby reducing neuroinflammation and triggering an anti‐inflammatory response within the brain (Fig. 2). For example, green tea catechins have been reported to reduce the production of iNOS and the expression of pro‐inflammatory markers (NFkB, IL6, TNF) therefore alleviating inflammation in the CNS [69]. Furthermore, (−)‐epicatechin (1 mg·kg^−1^ per day for 4 weeks) was reported to repress the activation of microglia and astrocytes therefore reducing the levels of pro‐inflammatory mediators (TNF‐α, IFN‐γ, IL‐1β) as well as promoting the secretion of anti‐inflammatory cytokines (IL‐10 and IL‐11) in the hippocampus from ageing mice [70]. In LPS‐induced mice, hesperetin (50 mg·kg^−1^ of body weight for 5 weeks) was found to improve behavioural disorders and to suppress astrocyte and microglia activation via inhibiting the protein expression of TLR4 [71].

Beyond flavonoids, low molecular weight (LMW) phenolic acids derived from microbial catabolism of (poly)phenols, endogenous metabolism (e.g. dopamine derived DOPAC and homovanillic acid) and/or food matrix (e.g. ferulic acid, caffeic acid) have recently received attention in the context of neuroinflammation [72]. Although most of the current research has been conducted *in vitro* using glial and activated microglial cells, with limited evidence from animal or human studies, the main molecular targets of the LMW (poly)phenol metabolites include: (a) the inhibition of inflammatory cytokines, including TNF‐α, IL‐1β, IL‐6; (b) the inhibition of iNOS and subsequent nitric oxide (NO) production as a response to glial activation; (c) the downregulation of COX‐2 and downstream prostaglandins; and (c) a downregulating NF‐κB through the modulation of glial and neuronal signalling pathways (see Carregosa *et al*. for an extensive review [73]).

### Interindividual variability in (poly)phenols metabolism

One of the main issues in understanding and exploiting the potential of (poly)phenols in the prevention of neurodegenerative diseases is the heterogeneity which exists within the individual response to their consumption. Such variations mainly originate from differences in the bioavailability and metabolism of (poly)phenols [74, 75] and are influenced by several factors such as personal characteristics (sex, age, ethnicity), lifestyle factors (diet, smoking, physical activity), (patho)physiological status, genetic background and gut microbiota composition and metabolism [76, 77]. These factors affect the processes involved in the absorption, distribution, metabolism and excretion (ADME) of (poly)phenols, therefore having a role in determining the amount and type of phenolic metabolites in the circulation. For instance, recent studies have investigated the existence of metabolic phenotypes (aka metabotypes) defining different clusters of individuals based on differences in excreted amount and profile of (poly)phenols ADME [78, 79, 80]. Controlling individual differences through metabotyping approaches may, therefore, contribute to understanding the drivers of the existing variabilities and help devise stratified/personalised strategies to delay the onset of neurodegenerative diseases [81].

### (Poly)phenols as modulators of the gut‐microbiota‐brain axis

Only 5–10% of dietary (poly)phenols are absorbed in the small intestine, and the remaining accumulate in the colon, where they undergo deconjugation and degradation by the microbiota before being absorbed [82]. Once absorbed, phenolic compounds undergo phase II enzymatic metabolism to increase their hydrophilicity and be excreted through urine [10]. (Poly)phenols are metabolised by the microbiota into phenolic acids, which are absorbed as bioactive compounds that have many beneficial properties for the host. For example, anthocyanin metabolism produces protocatechuic acid (PCA), which has been reported to exert neuroprotective actions [83]. Other metabolites derived from anthocyanin conversion are, hippuric, phenylacetic, and phenylpropenoic acids, which were shown to modulate vascular reactivity and to reduce the expression of inflammatory mediators [84]. Cocoa flavan‐3‐ols are primarily converted into phase 2 metabolites, phenyl‐γ‐valerolactones (many different forms exist) and phenylvaleric acids, but only secondarily into phenylpropioic and benzoic acids [85, 86]. Interestingly, polar compounds such as phenyl‐γ‐valerolactones and benzoic acids have been reported to cross the blood–brain barrier and locate into the brain [87, 88].

Several studies have suggested that (poly)phenols can modulate the gut microbiota composition, improve gut barrier function, and exert anti‐inflammatory effects, thereby influencing the gut‐brain axis (GBA) regulation, a complex bidirectional interaction between the central nervous system (CNS), the autonomic nervous system (ANS), the enteric nervous system (ENS) and the hypothalamic pituitary adrenal (HPA) axis [89]. The mechanisms underlying this communication include the regulation of bile acids homeostasis, intestinal inflammation and brain functioning through the synthesis of neurotransmitter precursors along with the production of many other gut‐derived metabolites (indoles, cresols, TMA, etc.) [90]. For example, (poly)phenols can interact with the gut microbiota, leading to the production of metabolites such as short‐chain fatty acids (SCFAs) and neurotransmitters, which can, in turn, affect brain function and behaviour [91]. In particular, (poly)phenols have been shown to promote the growth of beneficial gut bacteria such as *Lactobacillus*, *Bifidobacterium, Akkermansia, Roseburia*, and *Faecalibacterium* spp. while inhibiting the growth of harmful bacteria [92]. By maintaining a healthy balance of gut microbiota, (poly)phenols may help reduce inflammation and oxidative stress in the gut, contributing to improved cognitive function and a lower risk of cognitive decline [93]. For example, (−)‐epicatechin was reported to mitigate anxiety‐related behaviour by restoring aberrant *Lactobacillus* and *Enterobacter* abundances triggered by a high‐fat diet and by increasing hippocampal BDNF, as well as restoring the glucocorticoid receptor, the mineralocorticoid receptor and 11β‐hydroxysteroid dehydrogenase type 1 (11β‐HSD1) expression [94]. Furthermore, quercetin‐3‐*O*‐glucuronide (50 mg·kg^−1^) alleviated cognitive deficit and toxicity in amyloid beta (1–42)‐induced AD‐like mice through modulation of the gut microbiota and increase in SCFA production [95].

## Conclusion

(Poly)phenols have the potential to modulate the gut‐brain axis regulation and influence cognitive function and decline through their interactions with gut microbiota and their anti‐inflammatory and antioxidant properties. Incorporating (poly)phenol‐rich foods into the diet may be a promising strategy for maintaining brain health and reducing the risk of cognitive decline. However, long‐term human studies assessing the impact of chronic (poly)phenol intake on cognitive function across the lifespan and the progression of neurodegenerative diseases are still lacking. In addition, intervention trials determining the optimal doses and forms of (poly)phenols for neuroprotection and cognitive enhancement, along with their underlying molecular mechanisms (genomics, proteomics, metabolomics) for optimal intake levels would be necessary before health claims and/or recommended daily intake could be made. Furthermore, the role of genetic and epigenetic factors in individual responses to (poly)phenol consumption should be further explored along with the potential differential effects of (poly)phenols based on sex, ethnicity, and other demographic factors at the community and population levels. By addressing these areas, future research can provide a more comprehensive understanding of how (poly)phenols may contribute to brain health and pave the way for effective and personalised dietary strategies to enhance cognitive function and prevent cognitive decline.

## Author contributions

TH and DV wrote the manuscript. MGP revised the manuscript. All authors agreed on the final version of the manuscript.
